# Benzene-1,3,5-tricarbonyl trichloride

**DOI:** 10.1107/S1600536812012895

**Published:** 2012-03-31

**Authors:** Xin Fan, Yanrong Wang, Chengzhi Jin, Longfei Jin

**Affiliations:** a College of Chemistry and Material Science, South-Central University for Nationalities, Wuhan 430074, People’s Republic of China

## Abstract

In the title molecule, C_9_H_3_Cl_3_O_3_, there are three short interactions involving the benzene H atoms and the chloro­formyl Cl atoms. In the crystal, mol­ecules stack along the *a* axis with no significant non-bonded inter­actions.

## Related literature

For the preparation of the title compound, see: Hamel *et al.* (1968 ▶). For applications of 1,3,5-tri(chloro­form­yl)benzene, see: Buch *et al.* (2008 ▶); Li *et al.* (2007 ▶). For related structures and hydrogen bonding, see: Leser & Rabinovich (1978*a* ▶,*b* ▶); Jeffrey *et al.* (1985 ▶).
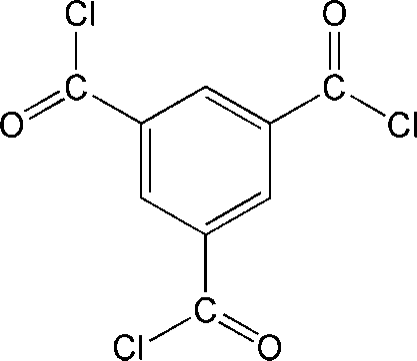


## Experimental

### 

#### Crystal data

C_9_H_3_Cl_3_O_3_
*M* *_r_* = 265.46
Orthorhombic, 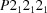

*a* = 6.0230 (13) Å
*b* = 8.3306 (18) Å
*c* = 21.314 (5) Å
*V* = 1069.4 (4) Å^3^
*Z* = 4
Mo *K*α radiation
μ = 0.84 mm^−1^
*T* = 298 K
0.30 × 0.20 × 0.20 mm

#### Data collection

Bruker SMART CCD area-detector diffractometer
Absorption correction: multi-scan (*SADABS*; Sheldrick, 1996 ▶) *T*_min_ = 0.787, *T*_max_ = 0.851
6552 measured reflections
2338 independent reflections
2267 reflections with *I* > 2σ(*I*)
*R*_int_ = 0.088

#### Refinement

*R*[*F* ^2^ > 2σ(*F* ^2^)] = 0.065
*wR*(*F* ^2^) = 0.170
*S* = 1.22
2338 reflections
136 parameters
H-atom parameters constrained
Δρ_max_ = 0.31 e Å^−3^
Δρ_min_ = −0.28 e Å^−3^
Absolute structure: Flack (1983 ▶), 952 Friedel pairs
Flack parameter: 0.13 (15)

### 

Data collection: *SMART* (Bruker, 2001 ▶); cell refinement: *SAINT-Plus* (Bruker, 2001 ▶); data reduction: *SAINT-Plus*; program(s) used to solve structure: *SHELXS97* (Sheldrick, 2008 ▶); program(s) used to refine structure: *SHELXL97* (Sheldrick, 2008 ▶); molecular graphics: *SHELXTL* (Sheldrick, 2008 ▶); software used to prepare material for publication: *SHELXTL*.

## Supplementary Material

Crystal structure: contains datablock(s) global, I. DOI: 10.1107/S1600536812012895/vm2166sup1.cif

Structure factors: contains datablock(s) I. DOI: 10.1107/S1600536812012895/vm2166Isup2.hkl

Supplementary material file. DOI: 10.1107/S1600536812012895/vm2166Isup3.cml

Additional supplementary materials:  crystallographic information; 3D view; checkCIF report

## Figures and Tables

**Table 1:** Hydrogen-bond geometry (Å, °)

*D*—H⋯*A*	*D*—H	H⋯*A*	*D*⋯*A*	*D*—H⋯*A*
C2—H2⋯Cl1	0.93	2.64	3.047 (5)	107
C4—H4⋯Cl2	0.93	2.62	3.036 (4)	108
C6—H6⋯Cl3	0.93	2.62	3.037 (4)	108

**Fig. 1.**
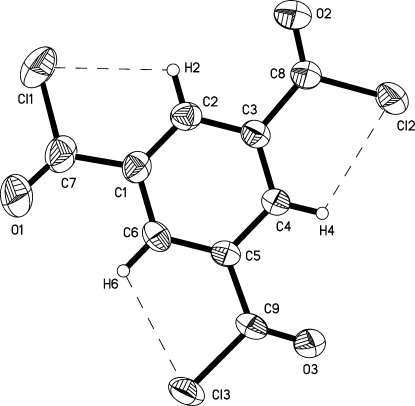
The molecular structure of (I), showing 30% probability displacement ellipsoids. Dashed lines indicate hydrogen bonds.

## supplementary crystallographic information

### Comment

1,3,5-Tri(chloroformyl)benzene is a commonly used monomer, mainly used in the preparation of reverse osmosis membranes and polyamide nanofiltration membranes (Buch *et al.*, 2008; Li *et al.*, 2007), which has good application prospects in the rapid development of today's film industry. As part of our ongoing studies, the preparation and X-ray structure determination of the title compound, (I), was undertaken.

The molecular structure of the title compound is shown in Figure 1. Bond lengths and angles in (I) show normal values. The geometric parameters of the title molecule agree well with those reported for similar structures (Leser & Rabinovich, 1978*a*,*b*). The non-hydrogen atoms of the molecule lie in a nearly plane with an r.m.s deviation of 0.1118 Å. Three intramolecular hydrogen bonds are observed between the C—H of phenyl group and the Cl atom of the chloroformyl group (Jeffrey *et al.*, 1985) (Figure 1 and Table 1). The shortest contacts in the crystal packing are O1···C2^i^ [3.094 (6) Å], O2···Cl3^ii^ [3.191 (5) Å] and O3···C9^iii^ [3.216 (6) Å] [symmetry code (i): -*x*,1/2 + *y*,1/2 - *z*; (ii): 1 + *x*,1 + *y*,*z*; (iii): 1/2 + *x*,3/2 - *y*,-*z*].

### Experimental

Compound (I) was synthesized according to the literature procedure of Hamel *et al.* (1968). Single crystals suitable for X-ray diffraction were grown by slow evaporation from 4:1 (V/V) mixed solution of petroleum ether and chloroform at room temperature.

### Refinement

All H atoms were included in the riding-model approximation, with C—H distances of 0.93 Å, and the isotropic displacement parameters were set equal to 1.2*U*_eq_ of the carrier atom.

### Crystal data

**Table:**

C_9_H_3_Cl_3_O_3_	*F*(000) = 528
*M**_r_* = 265.46	*D*_x_ = 1.649 Mg m^−^^3^
Orthorhombic, *P*2_1_2_1_2_1_	Mo *K*α radiation, λ = 0.71073 Å
Hall symbol: P 2ac 2ab	Cell parameters from 3613 reflections
*a* = 6.0230 (13) Å	θ = 2.4–28.2°
*b* = 8.3306 (18) Å	µ = 0.84 mm^−^^1^
*c* = 21.314 (5) Å	*T* = 298 K
*V* = 1069.4 (4) Å^3^	Block, colorless
*Z* = 4	0.30 × 0.20 × 0.20 mm

### Data collection

**Table:**

Bruker SMART CCD area-detector diffractometer	2338 independent reflections
Radiation source: fine-focus sealed tube	2267 reflections with *I* > 2σ(*I*)
Graphite monochromator	*R*_int_ = 0.088
phi and ω scans	θ_max_ = 27.0°, θ_min_ = 2.6°
Absorption correction: multi-scan (*SADABS*; Sheldrick, 1996)	*h* = −7→5
*T*_min_ = 0.787, *T*_max_ = 0.851	*k* = −9→10
6552 measured reflections	*l* = −27→27

### Refinement

**Table:**

Refinement on *F*^2^	Secondary atom site location: difference Fourier map
Least-squares matrix: full	Hydrogen site location: inferred from neighbouring sites
*R*[*F*^2^ > 2σ(*F*^2^)] = 0.065	H-atom parameters constrained
*wR*(*F*^2^) = 0.170	*w* = 1/[σ^2^(*F*_o_^2^) + (0.0522*P*)^2^ + 0.5279*P*] where *P* = (*F*_o_^2^ + 2*F*_c_^2^)/3
*S* = 1.22	(Δ/σ)_max_ < 0.001
2338 reflections	Δρ_max_ = 0.31 e Å^−^^3^
136 parameters	Δρ_min_ = −0.28 e Å^−^^3^
0 restraints	Absolute structure: Flack (1983), 952 Friedel pairs
Primary atom site location: structure-invariant direct methods	Flack parameter: 0.13 (15)

### Special details

**Table:**

Geometry. All e.s.d.'s (except the e.s.d. in the dihedral angle between two l.s. planes) are estimated using the full covariance matrix. The cell e.s.d.'s are taken into account individually in the estimation of e.s.d.'s in distances, angles and torsion angles; correlations between e.s.d.'s in cell parameters are only used when they are defined by crystal symmetry. An approximate (isotropic) treatment of cell e.s.d.'s is used for estimating e.s.d.'s involving l.s. planes.
Refinement. Refinement of *F*^2^ against ALL reflections. The weighted *R*-factor *wR* and goodness of fit *S* are based on *F*^2^, conventional *R*-factors *R* are based on *F*, with *F* set to zero for negative *F*^2^. The threshold expression of *F*^2^ > σ(*F*^2^) is used only for calculating *R*-factors(gt) *etc*. and is not relevant to the choice of reflections for refinement. *R*-factors based on *F*^2^ are statistically about twice as large as those based on *F*, and *R*- factors based on ALL data will be even larger.

### Fractional atomic coordinates and isotropic or equivalent isotropic displacement parameters (Å^2^)

**Table:**

	*x*	*y*	*z*	*U*_iso_*/*U*_eq_	
C1	0.4413 (7)	0.7665 (6)	0.83052 (18)	0.0582 (10)	
C2	0.3612 (7)	0.9176 (6)	0.84269 (18)	0.0582 (9)	
H2	0.2326	0.9534	0.8230	0.070*	
C3	0.4728 (7)	1.0170 (5)	0.88445 (19)	0.0545 (9)	
C4	0.6638 (6)	0.9669 (5)	0.91364 (18)	0.0497 (8)	
H4	0.7392	1.0352	0.9408	0.060*	
C5	0.7435 (6)	0.8124 (5)	0.90207 (17)	0.0482 (8)	
C6	0.6328 (7)	0.7132 (5)	0.86085 (19)	0.0553 (9)	
H6	0.6860	0.6102	0.8532	0.066*	
C7	0.3370 (9)	0.6507 (8)	0.7862 (3)	0.0834 (16)	
C8	0.3650 (9)	1.1749 (6)	0.8981 (3)	0.0764 (14)	
C9	0.9471 (7)	0.7645 (5)	0.9372 (2)	0.0565 (9)	
Cl1	0.1090 (3)	0.7279 (3)	0.74344 (9)	0.1188 (7)	
Cl2	0.5232 (3)	1.30825 (15)	0.94387 (9)	0.0934 (5)	
Cl3	1.0317 (2)	0.56293 (13)	0.92527 (7)	0.0775 (4)	
O1	0.3901 (9)	0.5166 (6)	0.7783 (2)	0.1201 (18)	
O2	0.1848 (8)	1.2126 (6)	0.8830 (3)	0.130 (2)	
O3	1.0513 (6)	0.8462 (4)	0.97113 (18)	0.0759 (9)	

### Atomic displacement parameters (Å^2^)

**Table:**

	*U*^11^	*U*^22^	*U*^33^	*U*^12^	*U*^13^	*U*^23^
C1	0.058 (2)	0.072 (3)	0.0452 (17)	0.0009 (19)	0.0046 (17)	−0.0110 (19)
C2	0.058 (2)	0.066 (2)	0.0502 (19)	0.008 (2)	−0.0014 (17)	0.0023 (19)
C3	0.058 (2)	0.0495 (19)	0.056 (2)	0.0058 (17)	0.0010 (18)	0.0014 (16)
C4	0.0481 (17)	0.0480 (18)	0.0530 (18)	0.0037 (15)	0.0025 (15)	0.0009 (16)
C5	0.0486 (18)	0.0464 (18)	0.0495 (18)	0.0055 (16)	0.0044 (15)	0.0063 (16)
C6	0.060 (2)	0.048 (2)	0.058 (2)	0.0056 (17)	0.0147 (18)	−0.0070 (17)
C7	0.076 (3)	0.103 (4)	0.070 (3)	0.003 (3)	−0.002 (2)	−0.034 (3)
C8	0.082 (3)	0.058 (2)	0.090 (3)	0.018 (2)	−0.029 (3)	−0.009 (2)
C9	0.0507 (19)	0.0460 (19)	0.073 (2)	0.0127 (16)	0.0077 (18)	0.0032 (19)
Cl1	0.1086 (11)	0.1431 (17)	0.1046 (11)	−0.0062 (12)	−0.0484 (10)	−0.0267 (12)
Cl2	0.0915 (9)	0.0520 (6)	0.1365 (13)	0.0142 (6)	−0.0241 (9)	−0.0208 (7)
Cl3	0.0730 (7)	0.0567 (6)	0.1028 (9)	0.0245 (5)	−0.0030 (6)	−0.0003 (6)
O1	0.123 (3)	0.112 (4)	0.125 (4)	0.020 (3)	−0.018 (3)	−0.076 (3)
O2	0.112 (3)	0.093 (3)	0.185 (5)	0.051 (3)	−0.071 (3)	−0.041 (3)
O3	0.0646 (18)	0.0658 (19)	0.097 (2)	0.0090 (15)	−0.0232 (18)	−0.0087 (19)

### Geometric parameters (Å, º)

**Table:**

C1—C2	1.373 (6)	C5—C6	1.378 (6)
C1—C6	1.394 (6)	C5—C9	1.491 (5)
C1—C7	1.489 (7)	C6—H6	0.9300
C2—C3	1.388 (6)	C7—O1	1.174 (7)
C2—H2	0.9300	C7—Cl1	1.769 (6)
C3—C4	1.373 (6)	C8—O2	1.175 (6)
C3—C8	1.496 (6)	C8—Cl2	1.758 (5)
C4—C5	1.396 (5)	C9—O3	1.174 (5)
C4—H4	0.9300	C9—Cl3	1.773 (4)
			
C2—C1—C6	119.6 (4)	C4—C5—C9	116.1 (4)
C2—C1—C7	124.4 (4)	C5—C6—C1	120.3 (4)
C6—C1—C7	115.9 (4)	C5—C6—H6	119.8
C1—C2—C3	119.9 (4)	C1—C6—H6	119.8
C1—C2—H2	120.1	O1—C7—C1	126.3 (6)
C3—C2—H2	120.1	O1—C7—Cl1	118.9 (5)
C4—C3—C2	121.0 (4)	C1—C7—Cl1	114.8 (4)
C4—C3—C8	122.8 (4)	O2—C8—C3	125.6 (5)
C2—C3—C8	116.1 (4)	O2—C8—Cl2	118.9 (4)
C3—C4—C5	119.2 (4)	C3—C8—Cl2	115.4 (3)
C3—C4—H4	120.4	O3—C9—C5	126.4 (4)
C5—C4—H4	120.4	O3—C9—Cl3	118.9 (3)
C6—C5—C4	119.9 (4)	C5—C9—Cl3	114.7 (3)
C6—C5—C9	123.9 (4)		
			
C6—C1—C2—C3	0.8 (6)	C2—C1—C7—O1	−173.6 (6)
C7—C1—C2—C3	−179.3 (4)	C6—C1—C7—O1	6.4 (9)
C1—C2—C3—C4	0.5 (6)	C2—C1—C7—Cl1	5.3 (7)
C1—C2—C3—C8	−175.9 (4)	C6—C1—C7—Cl1	−174.8 (3)
C2—C3—C4—C5	−1.5 (6)	C4—C3—C8—O2	−166.4 (7)
C8—C3—C4—C5	174.7 (4)	C2—C3—C8—O2	10.0 (9)
C3—C4—C5—C6	1.2 (6)	C4—C3—C8—Cl2	10.6 (6)
C3—C4—C5—C9	−177.8 (3)	C2—C3—C8—Cl2	−173.0 (4)
C4—C5—C6—C1	0.1 (6)	C6—C5—C9—O3	177.2 (4)
C9—C5—C6—C1	179.0 (4)	C4—C5—C9—O3	−3.8 (6)
C2—C1—C6—C5	−1.1 (6)	C6—C5—C9—Cl3	−3.1 (5)
C7—C1—C6—C5	179.0 (4)	C4—C5—C9—Cl3	175.9 (3)

### Hydrogen-bond geometry (Å, º)

**Table:**

*D*—H···*A*	*D*—H	H···*A*	*D*···*A*	*D*—H···*A*
C2—H2···Cl1	0.93	2.64	3.047 (5)	107
C4—H4···Cl2	0.93	2.62	3.036 (4)	108
C6—H6···Cl3	0.93	2.62	3.037 (4)	108
